# Mycoplasma genitalium molecular typing in men with non-gonococcal urethritis discriminates between phylogenetic clusters based on sexual preference and antibiotic resistance

**DOI:** 10.1099/jmm.0.001999

**Published:** 2025-04-25

**Authors:** Nikki Adriaens, Fenna M. Bouwman, Sylvia M. Bruisten, Clarissa E. Vergunst, Alje P. van Dam, Tessa A. Doelman, Brenda M. Westerhuis

**Affiliations:** 1 Department of Infectious Diseases, Public Health Service of Amsterdam, Amsterdam, Netherlands; 2 Amsterdam Institute for Immunology and Infectious Diseases, Amsterdam, Netherlands; 3 Department of Dermatology, NWZ, Den Helder, Netherlands; 4 Department of Medical Microbiology, Amsterdam UMC, Location University of Amsterdam, Amsterdam, Netherlands

**Keywords:** antibiotic resistance, *MG309*, *mgpB*, molecular epidemiology, molecular typing, *Mycoplasma genitalium*

## Abstract

**Introduction.***Mycoplasma genitalium* is a sexually transmitted bacterium associated with non-gonococcal urethritis (NGU) in men. The rising macrolide and fluoroquinolone resistance in *M. genitalium* has become a public health concern, requiring close surveillance.

**Gap statement.***MgpB/MG309* typing is commonly used to study genotype distribution and resistance patterns of *M. genitalium* in men who have sex with men (MSM); however, data for men who have sex with women (MSW) are limited.

**Aim.** The aim of this study was to explore the epidemiology of *M. genitalium* based on *mgpB*/*MG309* molecular typing in isolates from men diagnosed with NGU, comparing MSM and MSW. Additionally, antibiotic resistance was evaluated to assess associations between the *mgpB/MG309* genotypes, antimicrobial resistance profiles, and epidemiological determinants.

**Methodology.** A subset of previously collected *M. genitalium* isolates from men diagnosed with NGU in Amsterdam, the Netherlands, between May 2018 and November 2019 was analysed. Molecular typing was performed by sequencing relevant regions of the *mgpB* and *MG309* loci. Macrolide resistance was assessed by detecting mutations in the 23S rRNA gene via quantitative polymerase chain reaction, while fluoroquinolone resistance was determined through sequencing *parC* and *gyrA*.

**Results.** A total of 62 *M*. *genitalium* samples were analysed from 33 MSM and 29 MSW. The overall macrolide and fluoroquinolone resistance was 75.8% and 24.2 %, respectively. At the *mgpB* locus, 24 sequence types (STs) were identified, with ST4 most prevalent in MSM and ST2 in MSW. The *MG309* locus revealed 12 distinct short tandem repeat numbers, with repeat 10 being most common in both groups. Phylogenetic analysis based on *mgpB* sequences revealed two clusters: cluster A included significantly more MSW, whereas cluster B predominantly comprised MSM (*P*<0.001). Macrolide and fluoroquinolone resistance was significantly higher in cluster B compared with cluster A (*P*<0.01 and *P*<0.05, respectively).

**Conclusion.** Molecular typing of *M. genitalium* revealed two clusters that differed by sexual preference and antibiotic resistance, highlighting the importance of surveillance of resistance across genotypes. The findings suggest multiclonal spread of resistance through independent mutations. Future studies using next-generation sequencing are needed to further explore the links between sexual transmission and genetic diversity in *M. genitalium*.

## Data Availability

Data are available upon reasonable request. The datasets used and/or analysed during the current study are available from the corresponding author on reasonable request.

## Introduction

*Mycoplasma genitalium* is a sexually transmitted bacterium implicated in both acute and persistent non-gonococcal urethritis (NGU) in men [1]. The prevalence of *M. genitalium* infection among men diagnosed with NGU is ~10–35% [2], making it the second most common cause after *Chlamydia trachomatis* infection [3]. Due to its minimalistic genome and lack of a cell wall, *M. genitalium* exhibits intrinsic resistance to several antibiotics, limiting the options for treatment [1]. For symptomatic * M. genitalium* infections in men, current treatment guidelines in Europe recommend macrolides as the first-line antibiotic, followed by fluoroquinolones as the preferred alternative in case of initial therapy failure [4]. However, the extensive use of these antibiotics has led to widespread resistance in Europe, with the prevalence of macrolide and fluoroquinolone resistance mutations ranging from 8% to 89% and 4% to 34%, respectively [5].

Macrolide treatment failure is attributed to single nucleotide polymorphisms (SNPs) in domain V of the 23S rRNA gene at positions A2058 and A2059, referred to as macrolide resistance-associated mutations (MRAMs) [5,6]. Similarly, fluoroquinolone treatment failure is linked to mutations in the *parC* and *gyrA* genes, collectively known as the quinolone resistance-associated mutations (QRAMs). Clinically significant mutations include S83I, S83R, D87N and D87Y in *parC*, and M95I and D99Y in *gyrA*.^5^

To further explore the transmission dynamics of *M. genitalium* within populations, molecular typing methods are frequently employed. These include SNP analysis of the *mgpB* gene, which encodes the hypervariable adhesin protein MgPa [7], and the examination of the *MG309* gene, which involves quantifying the number of short tandem repeats (STRs), specifically the AGT/AAT motifs [8,9]. Combined, these analyses possess sufficient discriminatory power to effectively distinguish between closely related *M. genitalium* isolates [10]. While the *mgpB*/*MG309* molecular typing method has been commonly used to investigate *M. genitalium* transmission in sexual networks of men who have sex with men (MSM) [11,12] and women [11], only limited molecular typing data are available for *M. genitalium* isolates infecting men who have sex with women (MSW).

A comprehensive understanding of the distribution of *M. genitalium* genotypes across various populations and their potential association with resistance patterns is essential to combat the growing antimicrobial resistance observed. The aim of this study was to explore the epidemiology based on *mgpB*/*MG309* molecular typing among *M. genitalium* isolates from men diagnosed with NGU, comparing MSM and MSW. Additionally, antibiotic resistance was evaluated to assess potential associations between the *mgpB/MG309* genotypes, antimicrobial resistance profiles, and relevant epidemiological determinants.

## Methods

### Study patient population and sample selection

A subset of samples from a previously conducted study at the sexually transmitted infection (STI) clinic in Amsterdam, the Netherlands, was used [13]. From May 2018 to November 2019, men diagnosed with urethritis visiting the STI clinic were included. First-void urines were tested for *Neisseria gonorrhoeae, C. trachomatis* and *M. genitalium* using transcription-mediated amplification assays (Aptima, Hologic Inc, San Diego, CA, USA). *M. genitalium* infection was re-evaluated by *MgPa* quantitative polymerase chain reaction (qPCR) and genotyped for MRAM SNPs by single-target qPCR assays [14,15]. The subset of samples included in the current study consisted of randomly selected urine specimens that tested positive for *M. genitalium*, with a Ct-value <32.

### DNA sequencing and molecular typing

Sanger sequencing of relevant regions of *parC* (319 bp) and *gyrA* (228 bp) was performed on remnant DNA using primer combinations as previously described by Deguchi *et al.* [16]. The cycling conditions for *parC* and *gyrA* were as follows: activation step of 95 °C for 2 min; 35 cycles of 95 °C (15 s), 65 °C (30 s) and 72 °C (30 s); final elongation step of 72 °C for 6 min. Sanger sequencing of relevant regions of *mgpB* (281 bp) and *MG309* (~380 bp) was performed using primer combinations as previously described [7,8, 17]. Cycling conditions for *mgpB* were as follows: activation step of 98 °C for 30 s; second activation step of 95 °C for 2 min; 35 cycles of 95 °C (30 s), 60 °C (30 s) and 72 °C (60 s). For *MG309*, cycling conditions included: activation step of 95 °C for 15 min; 35 cycles of 95 °C (45 s), 56.8 °C (60 s) and 72 °C (60 s); final elongation step of 72 °C for 10 min.

PCR products were purified using the QIAquick PCR purification kit (Qiagen, Hilden, Germany). Subsequently, samples were sent for high-throughput Sanger sequencing to Eurofins Genomics (Ebersberg, Germany). Sequence analysis was performed using Bionumerics Version 7.6.3. (Applied Maths, BioMérieux, Marcy-l'Étoile, France). Obtained sequences were compared to the *M. genitalium* G37 reference strain (accession number NC_000908.2). For *MG309,* typing was determined by the number of STRs, independent of the specific repeat motifs, as is outlined in previously conducted studies [9,18]. Sequence typing of *mgpB* was based on SNPs, using the Public databases for molecular typing and microbial genome diversity (PubMLST) [19]. Newly found sequences were deposited to the PubMLST database, and consecutive sequence type (ST) numbering was given for isolates differing from the previously known genotypes. For *mgpB,* a maximum-likelihood phylogenetic tree using the best-fitting TPM2u+F+I substitution model and 1,000 bootstrap values was generated by IQ-TREE (version 2.3.4). Tree annotation was performed using iTol (version 6.9). The individual and combined discriminatory index (DI) for the *mgpB* and *MG309* typing methods was calculated by applying Simpson’s index of diversity [20].

### Statistical analysis

Patient information was extracted from electronic patient files which were blinded for the investigators. The variable ‘educational level’ was categorized into four groups as follows: low (no education, primary school, lower secondary vocational education, and intermediate secondary general education), middle (higher secondary general education, senior secondary vocational education, and pre-university secondary education), high (higher professional or university education) and unknown. Sexual preference was self-reported by the patients. Bisexual men were excluded from this study. The Chi-square test, Fisher’s exact test and Mann–Whitney U test were used to evaluate significant differences in epidemiological factors between MSM and MSW (*P*-value < 0.05). Statistical analyses were performed in SPSS Statistics (version 26.0, IBM, Armonk, NY, USA), and GraphPad Prism (version 8, GraphPad Software, La Jolla California, USA) was used to create figures.

## Results

### Patient characteristics

Sequencing was performed on *M. genitalium-*positive specimens originating from 126 patients diagnosed with NGU, successfully achieving sequencing for all four targets in isolates from 66 (52.4%) patients. Four of the patients were excluded since no sociodemographic and/or clinical data were available, leaving 62 (49.2%) patients for further analyses. Of these, 33 (53.2%) self-identified as MSM and 29 (46.8%) as MSW (Table 1). The median age of all patients was 27 years (interquartile range, 23–32.5), with MSM being significantly older compared with MSW (*P*<0.001). The majority reported their region of origin as either the Netherlands (37.1%) or Central and South America (35.5%). MSM had a higher proportion of patients originating from Asia, whereas MSW exhibited a significantly greater representation from Central and South America (*P*<0.05). Human immunodeficiency virus (HIV) status was available for 54/62 (87.1%) patients, with 9 (14.5%) testing positive, all of whom were MSM. Co-infections with *C. trachomatis* and *N. gonorrhoeae* were observed in 14.5% and 9.7% of all patients, respectively. MSW demonstrated a significantly higher prevalence of *C. trachomatis* co-infection (27.6%) compared with MSM (*P*<0.01). Additionally, a history of previous urethritis was reported by 48.5% of MSM, which was significantly higher than that in MSW (*P*<0.05). MRAMs were identified in 47 of 62 patients (75.8%), being 84.8% and 65.5% for MSM and MSW, respectively. The overall prevalence of QRAMs was observed in 24.2% of patients, with a higher prevalence of 30.3% in MSM. QRAMs were exclusively attributed to mutations in the *parC* gene, as no mutations in the *gyrA* gene were identified.

**Table 1.** Patient characteristics

	All patients (*n*=62)	MSM (*n*=33)	MSW (*n*=29)	*P-*value
**Age, median (IQR)**	27 (23–32.5)	30 (26–36.5)	24 (22.5–27.5)	<0.001^*^
**Region of origin,** ***n*** **(%)**				
The Netherlands	23 (37.1)	13 (39.4)	10(34.5)	<0.05†
Europe, outside the Netherlands	6 (9.7)	5 (15.2)	1 (3.4)	
Africa	4 (6.5)	2 (6.1)	2 (6.9)	
Asia	6 (9.7)	6 (18.2)	0 (0)	
Central and South America	22 (35.5)	7 (21.2)	15 (51.7)	
North America	0 (0)	0 (0)	0 (0)	
Oceania	0 (0)	0 (0)	0 (0)	
Unknown	1 (1.6)	0 (0)	1 (3.4)	
**HIV status,** ***n*** **(%)**				
Negative	45 (72.6)	23 (69.7)	22 (75.9	<0.001†
Positive	9 (14.5)	9 (27.3)	0 (0)	
Unknown	8 (12.9)	1 (3.0)	7 (24.1)	
**Education level,** ***n*** **(%)**				
Low	11 (17.7)	3 (9.1)	8 (27.6)	0.238†
Middle	11 (17.7)	6 (18.2)	5 (17.2)	
High	31 (50.0)	18 (54.5)	13 (44.8)	
Unknown	9 (14.6)	6 (18.2)	3 (10.3)	
**No. of sexual partners last 3 months, median (IQR)**	4.5 (2–10)	5 (3–14.5)	4 (2-7)	0.150^*^
***C. trachomatis*** **co-infection,** ***n*** **(%)**				
Negative	53 (85.5)	32 (97.0)	21 (72.4)	<0.01†
Positive	9 (14.5)	1 (3.0)	8 (27.6)	
***N. gonorrhoeae*** **co-infection,** ***n*** **(%)**				
Negative	56 (90.3)	29 (87.9)	27 (93.1)	0.679†
Positive	6 (9.7)	4 (12.1)	2 (6.9)	
**Previous antibiotic use last3 months,** ***n*** **(%)**				
No	46 (74.2)	21 (63.6)	25 (86.2)	0.079‡
Yes	16 (25.8)	12 (36.6)	4 (13.8)	
**Previous urethritis in last2 years,** ***n*** **(%)**				
No	40 (64.5)	17 (51.5)	23 (79.3)	<0.05‡
Yes	22 (35.5)	16 (48.5)	6 (20.7)	
**Macrolide resistance (MRAM),** ***n*** **(%)**				
Mutation	47 (75.8)	28 (84.8)	19 (65.5)	0.070*
Wildtype	15 (24.2)	5 (15.2)	10 (34.5)	
**Fluoroquinolone resistance (QRAM),** ***n*** **(%)**				
Mutation	15 (24.2)	10 (30.3)	5 (17.2)	0.254*
Wildtype	47 (75.8)	23 (69.7)	24 (82.8)	

*Mann–Whitney U test

†Fischer’s exact test

‡Chi-square test

HIV, human immunodeficiency virus; IQR, interquartile range; MRAM, macrolide resistance-associated mutation; MSM, men who have sex with men; MSW, men who have sex with women; QRAM, fluoroquinolone resistance-associated mutation.

### Molecular typing of *M. genitalium* isolates and antibiotic resistance

**Fig. 1.**
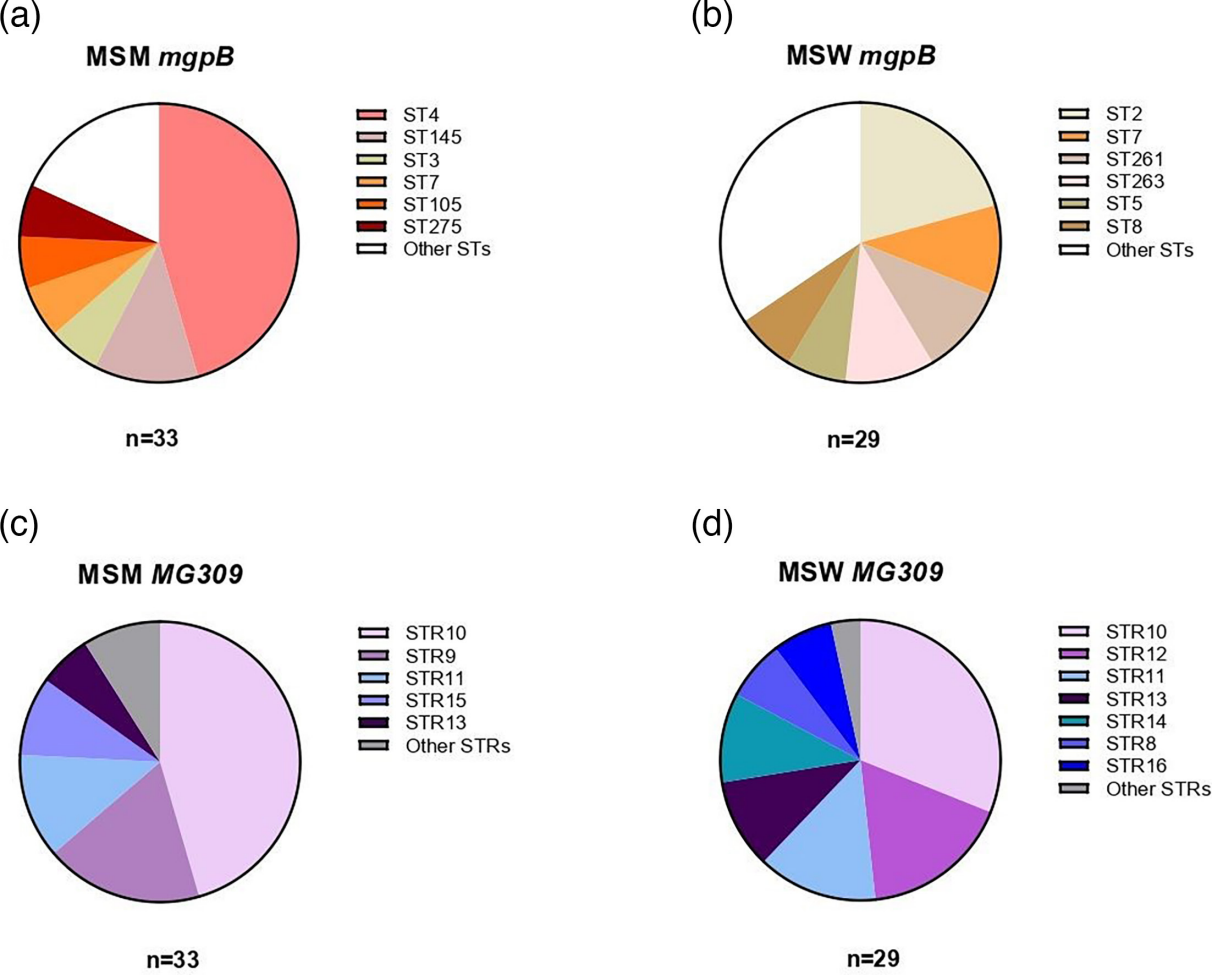
Distribution of most prevalent sequence types (STs) for *mgpB* in men who have sex with men (MSM) (**a**) and men who have sex with women (MSW) (**b**) and number of short tandem repeats (STRs) for *MG309* in MSM (**c**) and MSW (**d**). Only STs/STRs with a frequency count of ≥2 isolates are indicated.

**Fig. 2.**
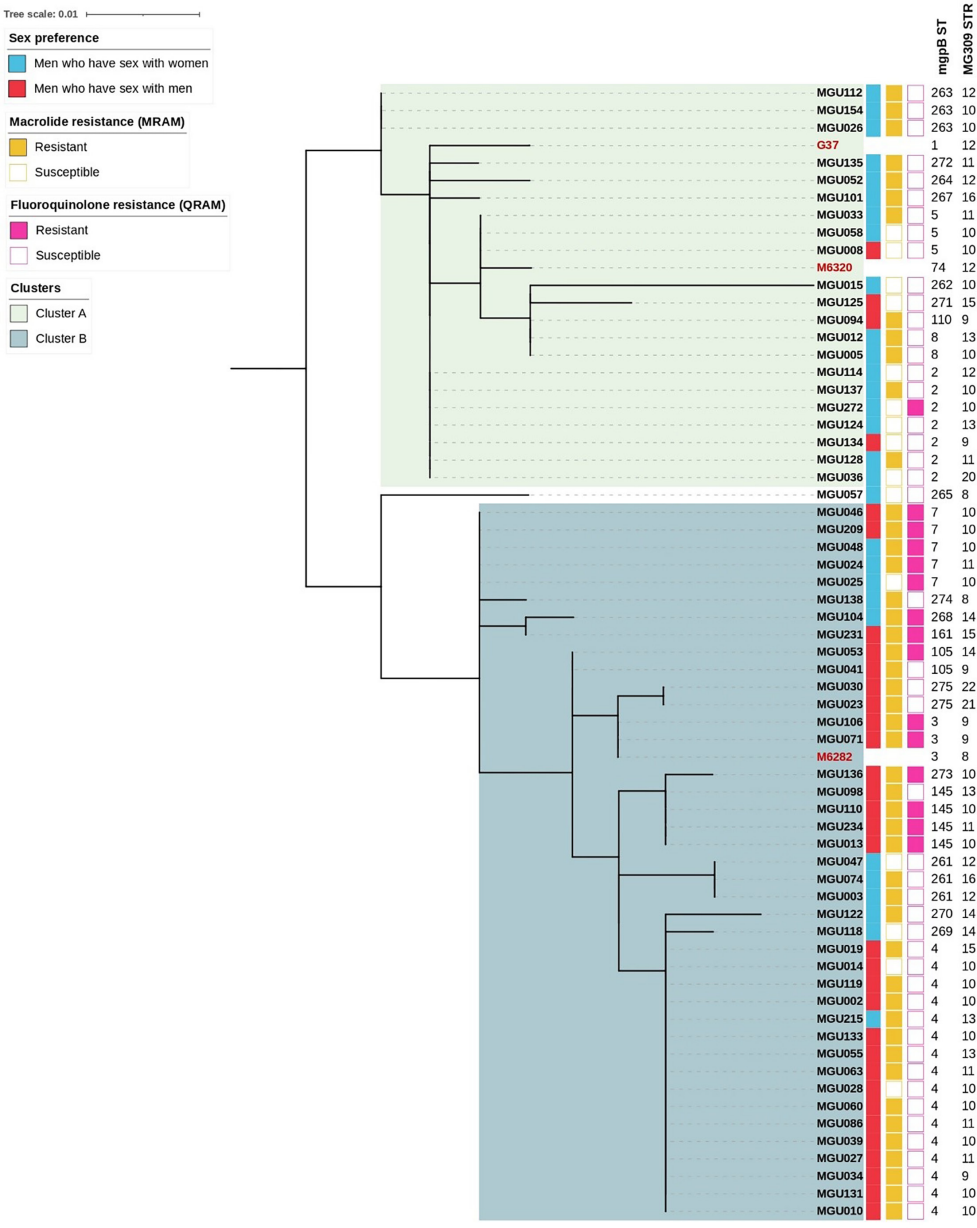
Phylogenetic tree of *mgpB* sequences from 62 *M. genitalium* isolates (black). Three previously documented isolates from PubMLST were included as references (red), including the reference strain (G37), M6282 (male with urethritis from Japan) and M6320 (male with urethritis from Australia). The tree is annotated with sample name, sex preference, macrolide resistance (MRAM), fluoroquinolone resistance (QRAM), *mgpB* sequence type (ST) and *MG309* short tandem repeat (STR) number. The maximum-likelihood tree was constructed using the TPM2u+F+I substitution method with 1,000 bootstraps. Clusters A and B could be identified, containing 21 and 40 *M. genitalium* isolates, respectively. Isolate MGU057 was designated as an outlier due to its low bootstrap value (Fig. S1) and was, therefore, excluded from the two phylogenetic clusters.

For the *mgpB* locus, 24 distinct STs were observed among the 62 patients included in this study (DI=0.9101), including 13 new STs (ST262–ST275) (Table S1, available in the online Supplementary Material). Of the 13 newly identified STs, 10 (76.9%) were isolated from patients in the MSW group. None of the observed STs matched the reference G37 strain (ST1). ST4 showed the highest prevalence among MSM, accounting for 45.5% of the cases, whereas ST2 was the most prevalent type among MSW, with a prevalence of 20.7% (Fig. 1a, b). Isolates with *mgpB* ST4 harboured MRAMs in 14 of 16 cases (87.5%), whereas none contained QRAMs (Fig. 2). In contrast, isolates with *mgpB* ST2 carried MRAMs in only 2/7 cases (28.6%) and QRAMs in 1/7 cases (14.3%). ST7 was among the most abundant ST for both MSM and MSW, with a prevalence of 6.1% and 10.3%, respectively. For *mgpB* ST7 isolates, MRAMs were present in 4/5 cases (80.0%) and QRAMs in all 5 cases (100%).

Analysis of the *MG309* locus identified 12 distinct numbers of STRs across the 62 patients included in this study (DI=0.8149), varying from 8 to 22 repeats (Table S2). Five isolates, all derived from MSW patients, exhibited an STR12, consistent with the reference G37 strain. For isolates from both MSM and MSW, STR10 was most prevalent, accounting for 45.5% and 31.0%, respectively (Fig. 1c–d). Isolates with STR10 contained MRAMs in 17 of 24 cases (70.8%), whereas isolates from 8 of 24 cases (33.3%) contained QRAMs (Fig. 2). The lowest STR number of 8 was observed in two MSW patients, corresponding to isolates MGU057 and MGU138. In contrast, the highest STRs of 21 and 22 were identified in isolates MGU030 and MGU023, both originating from MSM patients. Notably, these isolates shared the identical *mgpB* ST275.

The *mgpB*/*MG309* typing method identified 42 different genotypes in 62 *M. genitalium* isolates, resulting in a DI of 0.9736 (Table S3). Genotype 4.10, comprising ST4 and STR10, was highly prevalent among MSM, accounting for 27.3% of MSM cases. Isolates with genotype 4.10 contained MRAMs in 7 of 9 cases (77.8%), whereas none harboured QRAMs (Fig. 2). Otherwise, genotypes within both MSM and MSW did not exhibit any significant outlier; instead, the genotypes were relatively homogeneously distributed, with frequencies ranging from 0 to 3. The MSM and MSW groups shared only genotypes 4.13, 5.10 and 7.10, while all other genotypes were exclusive to either the MSM or MSW group.

### Phylogenetic clustering and epidemiological associations

The phylogenetic tree based on *mgpB* sequences (Fig. 2) revealed two distinct clusters among *M. genitalium* isolates. MGU057 was classified as an outlier due to low bootstrap values (Fig. S1), resulting in a cluster A comprising 21 isolates and a cluster B containing 40 isolates. The analysis of the association between these phylogenetic clusters and several epidemiological determinants (Table 2) showed that cluster A included a significantly higher proportion of MSW isolates, whereas cluster B was predominantly composed of isolates from MSM (*P*<0.001). The prevalence of MRAMs (*P*<0.01) and QRAMs (*P*<0.05) was significantly higher in cluster B (87.5% and 35.0%, respectively) compared with cluster A (57.1% and 4.8 %). Moreover, all 13 cases of dual-resistant isolates were observed in cluster B, significantly exceeding those in A, where none were observed (*P*<0.01). Finally, frequencies of the *mgpB* STs 2, 4, 5, 8 and 263 were significantly different between the two clusters (*P*<0.001). No statistically significant differences were noted between clusters in the frequency of specific point mutations for either MRAM or QRAM, type of sexual partner, region of origin, HIV status, *N. gonorrhoeae* co-infection, *C. trachomatis* co-infection, previous antibiotic use in the last 3 months, previous urethritis diagnosis in the last 2 years, or *MG309* STR.

**Table 2.** Differences in epidemiological determinants per phylogenetic cluster

	Cluster A (*n*=21)	Cluster B (*n*=40)	*P-value*
**Sex preference,** ***n*** **(%)**			<0.001*
Men who have sex with men	4 (19.0)	29 (72.5)	
Men who have sex with women	17 (81.0)	11 (27.5)	
**Type of sexual partner,** ***n*** **(%)**			0.552*
Casual partner	12 (57.1)	21 (52.5)	
Steady partner	2 (9.5)	8 (20.0)	
Both	7 (33.3)	11 (27.5)	
**Region of origin,** ***n*** **(%)**			0.052*
The Netherlands	8 (38.1)	14 (35.0)	
Europe, outside the Netherlands	0 (0)	7 (15.0)	
Africa	3 (14.3)	0 (0)	
Asia	0 (0)	6 (15.0)	
Central and South America	9 (42.9)	13 (32.5)	
North America	0 (0)	0 (0)	
Oceania	0 (0)	0 (0)	
Unknown	1 (4.8)	0 (0)	
**HIV status,** ***n*** **(%)**			0.649*
Negative	15 (71.4)	29 (72.5)	
Positive	2 (9.5)	7 (17.5)	
Unknown	4 (19.0)	4 (10.0)	
***N. gonorrhoeae*** **co-infection,** ***n*** **(%)**			0.688*
Negative	20 (95.2)	35 (87.5)	
Positive	1 (4.8)	5 (12.5)	
***C. trachomatis*** **co-infection,** ***n*** **(%)**			0.080*
Negative	15 (71.4)	37 (92.5)	
Positive	6 (28.6)	3 (7.5)	
**Previous antibiotic use last3 months,** ***n*** **(%)**			0.358*
No	18 (85.7)	27 (67.5)	
Yes	3 (14.3)	13 (32.5)	
**Previous urethritis in last2 years,** ***n*** **(%)**			0.067*
No	17 (81.0)	22 (55.0)	
Yes	4 (19.0)	18 (45.0)	
**Macrolide resistance (MRAM),** ***n*** **(%)**			<0.01*
Wildtype	9 (42.9)	5 (12.5)	
Mutation	12 (57.1)	35 (87.5)	
A2058G	3 (14.3)	10 (25.0)	
A2059G	6 (28.6)	24 (60.0)	
A2058C	0 (0)	0 (0)	
A2059C	0 (0)	0 (0)	
A2058T	3 (14.3)	1 (2.5)	
**Fluoroquinolone resistance (QRAM),** ***n*** **(%)**			<0.05*
Wildtype	20 (95.2)	26 (65.0)	
Mutation	1 (4.8)	14 (35.0)	
S83I	0 (0)	6 (15.0)	
S83R	0 (0)	0 (0)	
D87N	1 (4.8)	6 (15.0)	
D87Y	0 (0)	2 (5.0)	
**Dual MRAMandQRAM,** ***n*** **(%)**			<0.01*
No	21 (100)	27 (67.5)	
Yes	0 (0)	13 (32.5)	
***mgpB*** **ST**			<0.001*
2	7 (33.3)	0 (0)	
4	0 (0)	16 (40.4)	
5	3 (14.3)	0 (0)	
7	0 (0)	5 (12.5)	
145	0 (0)	4 (10.0)	
261	0 (0)	3 (7.5)	
263	3 (14.3)	0 (0)	
***MG309*** **STR**			0.849*
9	2 (9.5)	4 (10.0)	
10	8 (38.1)	16 (40.0)	
11	3 (14.3)	5 (12.5)	
12	3 (14.3)	2 (5.0)	
13	2 (9.5)	3 (7.5)	

*Fisher’s exact test. MGU057 was excluded from the two phylogenetic clusters. QRAM solely entails mutations in the parC gene. *MgpB* STs with frequency counts of ≥3 are shown. *MG309* STRs numbers with frequency counts of ≥5 are shown.

HIV, human immunodeficiency virus; MRAM, macrolide resistance-associated mutation; QRAM, fluoroquinolone resistance-associated mutation; ST, sequence type; STR, short tandem repeat.

## Discussion

This study aimed to explore the molecular epidemiology based on *mgpB*/*MG309* typing among *M. genitalium* isolates collected from men diagnosed with NGU, comparing two groups of MSM and MSW. In addition, antibiotic resistance was evaluated to investigate a potential link between genotypes, antimicrobial resistance profiles, and epidemiological determinants.

Based on *mgpB* typing only, our findings demonstrated two distinct phylogenetic clusters of *M. genitalium* isolates, with significant differences between clusters based on sexual preference and antibiotic resistance. Cluster A was predominantly composed of isolates from MSW (81.0%), whereas cluster B primarily consisted of isolates from MSM (72.5%), highlighting the significant difference in the distribution of sexual preference between the two clusters. This is in accordance with Guiraud *et al.* [11], who identified distinct genetic clustering in a large group of *M. genitalium* isolates from MSM and women. Three distinct clusters were observed based on *mgpB* STs, with one cluster showing a significantly higher proportion of MSM relative to the other clusters, which consisted predominantly of isolates from women. Moreover, Fernández-Huerta *et al.* [21] performed *mgpB* typing on 54 asymptomatic individuals and demonstrated two clusters, where one cluster significantly correlated with infections occurring in women and MSW, and a second cluster mostly entailed infections in bisexual men and MSM. Collectively, these findings suggest that *mgpB* typing can differentiate between distinct sexual networks of MSM and MSW. This association is not apparent for the *MG309* locus*,* considering the majority of samples in both clusters contained STR10. Including *MG309* could provide additional data on direct transmission routes, although, in this study, the added value is limited, possibly due to the sample size and lower genetic diversity.

In the *mgpB* phylogenetic tree, branch lengths for MSM isolates were notably longer compared with MSW, reflecting a higher genetic distance among MSM isolates. Several factors may have contributed to this, including the high levels of genetic recombination identified in *M. genitalium*. In populations with enhanced transmission rates, such as MSM, recombination events may occur more frequently, contributing to the increased genetic diversity [2,22]. Furthermore, the increased prevalence of antibiotic resistance in MSM populations may have created a stronger selective pressure on *M. genitalium* isolates, leading to more frequent mutations [23].

The overall prevalence of macrolide and fluoroquinolone resistance in the study population was 75.8% and 24.2%, respectively. The resistance of both macrolides and fluoroquinolones was significantly elevated in cluster B (87.5% and 35.0%, respectively), which predominantly comprised samples from MSM, compared with cluster A (57.1% and 4.8%, respectively). This is consistent with previous studies evaluating the prevalence of macrolide-resistant *M. genitalium* isolates across different populations [11,21, 23]. The high levels of resistance observed in this group could be attributed to the high transmission rate of resistant *M. genitalium* isolates within MSM communities but may also reflect the acquisition of resistance after increased antibiotic use [24,25]. This is particularly problematic in populations with increased risk of STIs, especially when individuals are regularly screened and treated with azithromycin-targeting pathogens other than *M. genitalium* [18,26]. To prevent further undesirable spread of macrolide resistance, some guidelines have recommended discontinuing azithromycin as part of the presumptive management of STIs [27,28].

This study identified 13/24 novel *mgpB* STs among the collected *M. genitalium* isolates, based on PubMLST. Ten new STs originated from MSW isolates, likely reflecting a sampling bias in existing *M. genitalium* ST databases, which predominantly include MSM isolates due to prior study focus. ST2 was the predominant *mgpB* ST within the MSW group, accounting for 20.7% of the cases. This ST exhibited notably lower levels of MRAMs and QRAMs, 28.6% and 14.3%, respectively, relative to ST4, ST7, and ST145. Similar findings have been reported by Guiraud *et al.* [11] who observed MRAMs in 7.4% and QRAMs in 24.1% of ST2 isolates. The *mgpB* ST7 was detected in both MSM and MSW groups. This ST exhibited high levels of multidrug resistance, with MRAMs and FRAMs recorded at 80% and 100 %, respectively. In contrast, previous studies have reported significantly lower resistance rates for ST7; however, the overall prevalence of this ST remains low across various studies [11,18].

Within the MSM group, *mgpB* ST4 was most common (45.5%), consistent with other studies reporting a dominance of ST4 within MSM populations [11,12, 18], suggesting the spread of a specific clone [18,21, 29]. The reason for this increased prevalence remains unknown; however, a hypothesis could be that the increased prevalence is attributed to the widespread occurrence of this type in a self-contained sexual network [21]. ST4 showed a high level of MRAM prevalence of 87.5%, while no FRAM mutations were detected. Other studies have equally demonstrated that ST4 often harbours MRAMs, while in contrast, FRAMs are rare [11,18].

While certain *mgpB* STs exhibit distinct trends in antibiotic resistance, these patterns may be more strongly associated with sexual preference than with inherent genotypic characteristics of *M. genitalium* isolates themselves. For instance, *mgpB* ST4 demonstrated elevated levels of macrolide resistance but was exclusively identified in MSM, a population with a documented higher burden of antibiotic resistance [11,21, 23]. Conversely, ST2, which displayed relatively low levels of antibiotic resistance, was predominantly observed in MSW, a group generally associated with lower antibiotic resistance profiles. This suggests that resistance is likely to arise through independent mutations, supporting previous findings that the development of antibiotic resistance in *M. genitalium* is multiclonal, with no strong correlation between genotypes and antibiotic resistance [7,22, 24, 30].

In contrast with previous studies, which primarily included *M. genitalium* isolates from asymptomatic individuals [11,12, 18, 21], this study focused exclusively on isolates from individuals diagnosed with NGU. Notably, the most frequently identified *mgpB* STs in this study were also reported in earlier studies that were not limited to symptomatic patients, suggesting that there is no apparent correlation to pathogenicity.

A strength of this study includes the comprehensive collection of epidemiological data, co-infections and genotypic analyses, which substantially strengthens the robustness of our findings. There are some limitations to our study that should be addressed. First, this study lacks detailed information on sexual interactions between patients, which constrained our ability to fully evaluate direct transmission between closely related isolates. Second, a group of women infected with *M. genitalium* was missing in order to elaborate on whether the isolates would closely cluster to the isolates originating from MSW.

In conclusion, we described the molecular typing and antibiotic resistance of *M. genitalium* isolates from MSM and MSW NGU patients in the Netherlands. Molecular typing revealed clusters differentiated by sexual preference and antibiotic resistance profiles. These findings provide evidence regarding the molecular epidemiology and transmission dynamics for *M. genitalium* infections within sexual networks, underlining the need to expand the monitoring of antibiotic resistance in these populations. Moreover, our data suggest that resistance is likely to arise through independent mutations, supporting the multiclonal spread of antibiotic resistance in *M. genitalium*. Future studies using next-generation sequencing are needed to further explore the relationship between sexual transmission and genetic diversity in *M. genitalium*.

## Supplementary material

10.1099/jmm.0.001999 Uncited Supplementary Material 1.

## Abbreviations

DI: discriminatory index
MRAMs: macrolide resistance-associated mutations
MSM: men who have sex with men
MSW: men who have sex with women
NGU: non-gonococcal urethritis
PubMLST: public databases for molecular typing and microbial genome diversity
qPCR: quantitative polymerase chain reaction
QRAMs: quinolone resistance-associated mutations
SNPs: single nucleotide polymorphisms
ST: sequence type
STI: sexually transmitted infection
STRs: short tandem repeats

## References

[1] Gnanadurai R, Fifer H (2020). Mycoplasma genitalium: a review. Microbiology.

[2] Taylor-Robinson D, Jensen JS (2011). *Mycoplasma genitalium*: from chrysalis to multicolored butterfly. Clin Microbiol Rev.

[3] Bradshaw CS, Tabrizi SN, Read TRH, Garland SM, Hopkins CA (2006). Etiologies of nongonococcal urethritis: bacteria, viruses, and the association with orogenital exposure. J Infect Dis.

[4] Jensen JS, Cusini M, Gomberg M, Moi H, Wilson J (2022). 2021 European guideline on the management of *Mycoplasma genitalium* infections. Acad Dermatol Venereol.

[5] Jensen JS, Unemo M (2024). Antimicrobial treatment and resistance in sexually transmitted bacterial infections. Nat Rev Microbiol.

[6] Jensen JS, Bradshaw CS, Tabrizi SN, Fairley CK, Hamasuna R (2008). Azithromycin treatment failure in *Mycoplasma genitalium*-positive patients with nongonococcal urethritis is associated with induced macrolide resistance. Clin Infect Dis.

[7] Hjorth SV, Björnelius E, Lidbrink P, Falk L, Dohn B (2006). Sequence-based typing of *Mycoplasma genitalium* reveals sexual transmission. J Clin Microbiol.

[8] Ma L, Martin DH (2004). Single-nucleotide polymorphisms in the rRNA operon and variable numbers of tandem repeats in the lipoprotein gene among *Mycoplasma genitalium* strains from clinical specimens. J Clin Microbiol.

[9] Ma L, Taylor S, Jensen JS, Myers L, Lillis R (2008). Short tandem repeat sequences in the *Mycoplasma genitalium* genome and their use in a multilocus genotyping system. BMC Microbiol.

[10] Dumke R (2022). Molecular tools for typing *Mycoplasma pneumoniae* and *Mycoplasma genitalium*. Front Microbiol.

[11] Guiraud J, Helary M, Le Roy C, Elguero E, Pereyre S (2022). Molecular typing reveals distinct *Mycoplasma genitalium* transmission networks among a cohort of men who have sex with men and a cohort of women in France. Microorganisms.

[12] Dumke R, Rust M, Glaunsinger T (2020). *mgpB* types among *Mycoplasma genitalium* strains from men who have sex with men in Berlin, Germany, 2016–2018. Pathogens.

[13] Braam JF, van Dam AP, Bruisten SM, van Rooijen MS, de Vries HJC (2022). Macrolide-resistant *Mycoplasma genitalium* impairs clinical improvement of male urethritis after empirical treatment. Sex Transm Dis.

[14] Jensen JS, Björnelius E, Dohn B, Lidbrink P (2004). Use of TaqMan 5’ nuclease real-time PCR for quantitative detection of *Mycoplasma genitalium* DNA in males with and without urethritis who were attendees at a sexually transmitted disease clinic. J Clin Microbiol.

[15] Braam JF, Hetem DJ, Vergunst CE, Kuizenga Wessel S, van Rooijen MS (2020). Evaluating the prevalence and risk factors for macrolide resistance in *Mycoplasma genitalium* using a newly developed qPCR assay. PLoS One.

[16] Deguchi T, Maeda S, Tamaki M, Yoshida T, Ishiko H (2001). Analysis of the gyrA and parC genes of *Mycoplasma genitalium* detected in first-pass urine of men with non-gonococcal urethritis before and after fluoroquinolone treatment. J Antimicrob Chemother.

[17] Jensen JS, Uldum SA, Søndergård-Andersen J, Vuust J, Lind K (1991). Polymerase chain reaction for detection of *Mycoplasma genitalium* in clinical samples. J Clin Microbiol.

[18] Guiraud J, Lounnas M, Boissière A, Le Roy C, Elguero E (2021). Lower *mgpB* diversity in macrolide-resistant Mycoplasma genitalium infecting men visiting two sexually transmitted infection clinics in Montpellier, France. J Antimicrob Chemother.

[19] Jolley K, Bray J, Maiden M (2018). Open-access bacterial population genomics: BIGSdb software, the PubMLST.org website and their applications. Wellcome Open Res.

[20] Hunter PR, Gaston MA (1988). Numerical index of the discriminatory ability of typing systems: an application of simpson’s index of diversity. J Clin Microbiol.

[21] Fernández-Huerta M, Serra-Pladevall J, Esperalba J, Moreno-Mingorance A, Fernández-Naval C (2020). Single-locus-sequence-based typing of the *mgpB* gene reveals transmission dynamics in *Mycoplasma genitalium*. J Clin Microbiol.

[22] Fookes MC, Hadfield J, Harris S, Parmar S, Unemo M (2017). *Mycoplasma genitalium*: whole genome sequence analysis, recombination and population structure. BMC Genomics.

[23] Machalek DA, Tao Y, Shilling H, Jensen JS, Unemo M (2020). Prevalence of mutations associated with resistance to macrolides and fluoroquinolones in *Mycoplasma genitalium*: a systematic review and meta-analysis. Lancet Infect Dis.

[24] Piñeiro L, Idigoras P, Cilla G (2019). Molecular typing of *Mycoplasma genitalium*-positive specimens discriminates between persistent and recurrent infections in cases of treatment failure and supports contact tracing. Microorganisms.

[25] Kenyon C, De Baetselier I, Vanbaelen T, Buyze J, Florence E (2021). The population-level effect of screening for *Mycoplasma genitalium* on antimicrobial resistance: a quasi-experimental study. Sex Transm Dis.

[26] Read TRH, Fairley CK, Murray GL, Jensen JS, Danielewski J (2019). Outcomes of resistance-guided sequential treatment of *Mycoplasma genitalium* infections: a prospective evaluation. Clin Infect Dis.

[27] Soni S, Horner P, Rayment M, Pinto-Sander N, Naous N (2019). British association for sexual health and HIV national guideline for the management of infection with *Mycoplasma genitalium* (2018). Int J STD AIDS.

[28] ASHM (2024). Australian STI management guidelines for use in primary care Mycoplasma genitalium.

[29] Dumke R, Spornraft-Ragaller P (2021). Antibiotic resistance and genotypes of *Mycoplasma genitalium* during a resistance-guided treatment regime in a German University Hospital. Antibiotics.

[30] Anagrius C, Loré B, Jensen JS (2013). Treatment of *Mycoplasma genitalium*. Observations from a Swedish STD clinic. PLoS One.

