# Supplementary material for: Mycoplasma genitalium molecular typing in men with non-gonococcal urethritis discriminates between phylogenetic clusters based on sexual preference and antibiotic resistance
Source: J Med Microbiol. 2025 Apr 25;74(4):001999. doi: 10.1099/jmm.0.001999 (PMC12032421; doi:10.1099/jmm.0.001999)
Supplement: Uncited Supplementary Material 1. [file jmm-74-01999-s001.pdf]

SUPPLEMENTARY DATA

Supplementary Table 1. *mgpB* sequence types

mgpB sequence type	All patients (n=62) n (%)	MSM (n=33) n (%)	MSW (n=29) n (%)
4	16 (25.8)	15 (45.5)	1 (3.5)
2	7 (11.3)	1 (3.0)	6 (20.7)
7	5 (8.1)	2 (6.1)	3 (10.3)
145	4 (6.5)	4 (12.1)	0 (0)
5	3 (4.8)	1 (3.0)	2 (6.9)
261	3 (4.8)	0 (0)	3 (10.3)
263*	3 (4.8)	0 (0)	3 (10.3)
3	2 (3.2)	2 (6.1)	0 (0)
8	2 (3.2)	0 (0)	2 (6.9)
105	2 (3.2)	2 (6.1)	0 (0)
275*	2 (3.2)	2 (6.1)	0 (0)
110	1 (1.6)	1 (3.0)	0 (0)
161	1 (1.6)	1 (3.0)	0 (0)
262*	1 (1.6)	0 (0)	1 (3.5)
264*	1 (1.6)	0 (0)	1 (3.5)
265*	1 (1.6)	0 (0)	1 (3.5)
267*	1 (1.6)	0 (0)	1 (3.5)
268*	1 (1.6)	0 (0)	1 (3.5)
269*	1 (1.6)	0 (0)	1 (3.5)
270*	1 (1.6)	0 (0)	1 (3.5)
271*	1 (1.6)	1 (3.0)	0 (0)
272*	1 (1.6)	0 (0)	1 (3.5)
273*	1 (1.6)	1 (3.0)	0 (0)
274*	1 (1.6)	0 (0)	1 (3.5)

MSM, men who have sex with men; MSW, men who have sex with women.*New sequence types were deposited to the PubMLST database.

Supplementary Table 2. *MG309* number of short tandem repeats

MG309 **number of STRs**	**All patients (n=62) n (%)**	**MSM (n=33) n (%)**	**MSW (n=29) n (%)**
10	24 (38.7)	15 (45.5)	9 (31.0)
11	8 (12.9)	4 (12.1)	4 (13.8)
9	6 (9.7)	6 (18.2)	0 (0)
12	5 (8.1)	0 (0)	5 (17.2)
13	5 (8.1)	2 (6.1)	3 (10.3)
14	4 (6.5)	1 (3.0)	3 (10.3)
15	3 (4.8)	3 (9.1)	0 (0)
8	2 (3.2)	0 (0)	2 (6.9)
16	2 (3.2)	0 (0)	2 (6.9)
20	1 (1.6)	0 (0)	1 (3.4)
21	1 (1.6)	1 (3.0)	0 (0)
22	1 (1.6)	1 (3.0)	0 (0)

STR, short tandem repeats; MSM, men who have sex with men; MSW, men who have sex with women.

Supplementary Table 3. Genotypes obtained through *mgpB*/*MG309* MLST

Genotype	*mgpB* sequence type	*MG309* number of STRs	All patients (n=62) n (%)	MSM (n=33) n (%)	MSW (n=29) n (%)
4.10	4	10	9 (14.5)	9 (27.3)	0 (0)
4.11	4	11	3 (4.8)	3 (9.1)	0 (0)
7.10	7	10	3 (4.8)	2 (6.1)	1 (3.5)
2.10	2	10	2 (3.2)	0 (0)	2 (6.9)
3.9	3	9	2 (3.2)	2 (6.1)	0 (0)
4.13	4	13	2 (3.2)	1 (3.0)	1 (3.5)
5.10	5	10	2 (3.2)	1 (3.0)	1 (3.5)
7.11	7	11	2 (3.2)	0 (0)	2 (6.9)
145.10	145	10	2 (3.2)	2 (6.1)	0 (0)
261.12	261	12	2 (3.2)	0 (0)	2 (6.9)
263.10	263	10	2 (3.2)	0 (0)	2 (6.9)
2.9	2	9	1 (1.6)	1 (3.0)	0 (0)
2.11	2	11	1 (1.6)	0 (0)	1 (3.5)
2.12	2	12	1 (1.6)	0 (0)	1 (3.5)
2.13	2	13	1 (1.6)	0 (0)	1 (3.5)
2.20	2	20	1 (1.6)	0 (0)	1 (3.5)
4.9	4	9	1 (1.6)	1 (3.0)	0 (0)
4.15	4	15	1 (1.6)	1 (3.0)	0 (0)
5.11	5	11	1 (1.6)	0 (0)	1 (3.5)
8.10	8	10	1 (1.6)	0 (0)	1 (3.5)
8.13	8	13	1 (1.6)	0 (0)	1 (3.5)
105.9	105	9	1 (1.6)	1 (3.0)	0 (0)
105.14	105	14	1 (1.6)	1 (3.0)	0 (0)
110.9	110	9	1 (1.6)	1 (3.0)	0 (0)
145.11	145	11	1 (1.6)	1 (3.0)	0 (0)
145.13	145	13	1 (1.6)	1 (3.0)	0 (0)
161.15	161	15	1 (1.6)	1 (3.0)	0 (0)
261.16	261	16	1 (1.6)	0 (0)	1 (3.5)
262.10	262	10	1 (1.6)	0 (0)	1 (3.5)
263.12	263	12	1 (1.6)	0 (0)	1 (3.5)
264.12	264	12	1 (1.6)	0 (0)	1 (3.5)
265.8	265	8	1 (1.6)	0 (0)	1 (3.5)
267.16	267	16	1 (1.6)	0 (0)	1 (3.5)
268.14	268	14	1 (1.6)	0 (0)	1 (3.5)
269.14	269	14	1 (1.6)	0 (0)	1 (3.5)
270.14	270	14	1 (1.6)	0 (0)	1 (3.5)
271.15	271	15	1 (1.6)	1 (3.0)	0 (0)
272.11	272	11	1 (1.6)	0 (0)	1 (3.5)
273.10	273	10	1 (1.6)	1 (3.0)	0 (0)
274.8	274	8	1 (1.6)	0 (0)	1 (3.5)
275.21	275	21	1 (1.6)	1 (3.0)	0 (0)
275.22	275	22	1 (1.6)	1 (3.0)	0 (0)

STRs, short tandem repeats; MSM, men who have sex with men; MSW, men who have sex with women.

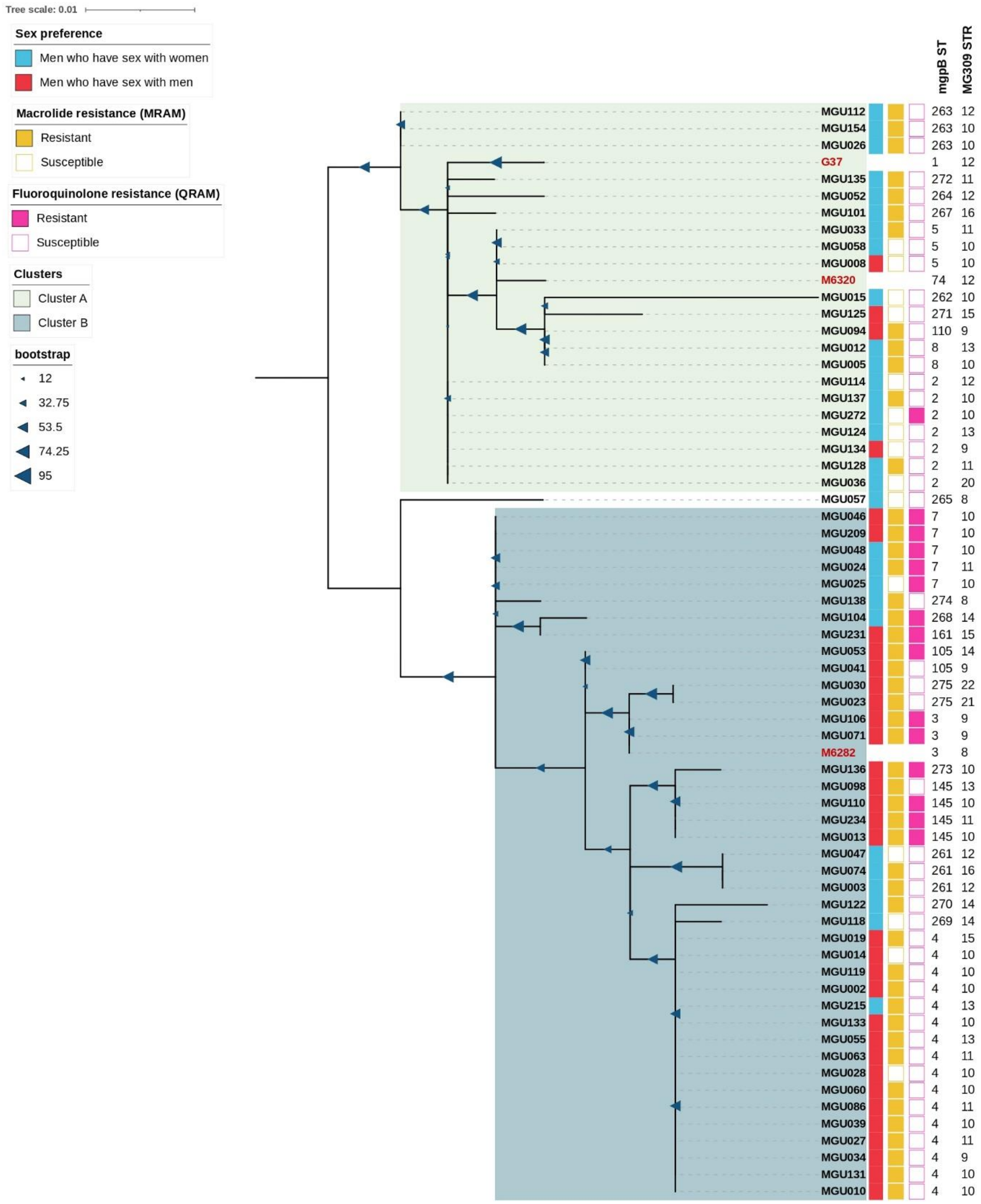

Supplementary Figure 1. Phylogenetic tree of the 62 *M. genitalium* isolates including bootstrap values
